# Supplementary material for: Symbiosis reshapes metabolism of sulfate-reducing bacteria in gutless marine worms
Source: ISME J. 2026 Jul 17;20(1):wrag190. doi: 10.1093/ismejo/wrag190 (PMC13548325; doi:10.1093/ismejo/wrag190)
Supplement: Supplementary file 1 [file supplementary_material_wrag190.zip › Supplemental_Material.pdf]

Supplementary Material:

Symbiosis reshapes metabolism of sulfate-reducing bacteria in gutless marine worms

Grace D'Angelo[1], Manuel Kleiner[2], Anna Mankowski[1,3], Jerónimo Cifuentes-Anticevic[1], Marlene J. Violette[4], Valerie De Anda[5], Marc Mussmann[6], Eileen Kröber[1], Nicole Dubilier[1,7*], Manuel Liebeke[1,8*]

[1]Max Planck Institute for Marine Microbiology, Bremen, Germany

[2]Department of Plant and Microbial Biology, North Carolina State University, Raleigh, NC, USA

[3]Molecuar Systems Biology Unit, European Molecular Biology Laboratory, Heidelberg, Germany

[4]Department of Biostatistics and Bioinformatics, Duke University School of Medicine, Durham, NC, USA

[5]Department of Functional and Evolutionary Ecology, Microbial Oceanography Unit, University of Vienna, Vienna, Austria

[6]Centre for Microbiology and Environmental Systems Science, Division of Microbial Ecology, University of Vienna, Vienna, Austria

[7]MARUM Center for Marine Environmental Sciences, University of Bremen, Bremen, Germany

[8]Institute of Human Nutrition and Food Science, Department of Metabolomics, University of Kiel, Kiel, Germany

*corresponding authors: mliebeke@mpi-bremen.de, ndubilie@mpi-bremen.de

Contents:

Supplementary text

Supplement Tables 1-12; provided in a separate file

Supplementary Figures 1-3

Supplementary text:

The core metabolism of the *Desulfoconcordia* symbionts is stable across host individuals

To complement the bulk metaproteomics approach, we reanalyzed published protein expression data from individual worms. While the metaproteomes generated from 50 worms allowed us to target low-abundance symbiont proteins, we also investigated potential variations in gene expression at the individual host level that might be masked by a bulk approach (**Fig. S3**). Overall, the top abundant proteins in *Desulfoconcordia* were similar across individual worms (**Table S12**, **Fig. S3**) and consistent with those in the bulk analysis (**Table S11**). In all *Desulfoconcordia* proteomes from single-host metaproteomes, the most highly expressed gene was a hypothetical protein, sharing approximately 50% sequence identity (e-value of 10^{-109}) with porin family proteins from members of the Desulfobacterota phylum in the NCBI BLAST database. Among the top 10 most abundant proteins were ABC transporters for amino acids and GroEL, a chaperone protein. Additionally, the anaerobic bidirectional form of carbon monoxide dehydrogenase of the Wood-Ljungdahl pathway was consistently within the top 100 proteins of single-host individuals. These complementary analyses provide a comprehensive understanding of *Desulfoconcordia* in *Olavius algarvensis* and a more detailed view of the variations in gene expression at the individual community level (**Fig. S3**).

Reanalysis of published data

Publicly available metaproteomic data were downloaded from the PRIDE (https://www.ebi.ac.uk/pride/) repository for reanalysis (Project ID: PXD014591)[1]. These mass spectrometry files were searched in Proteome Discoverer 2.5 against an updated metaproteomic reference database (OlaviusV13.fasta). The MS/MS spectra were searched against this reference database using the Sequest HT algorithm in Proteome Discoverer 2.5

(Thermo Fisher Scientific) with the following parameters: trypsin (full), maximum of two missed cleavages, 10 pp precursor mass tolerance, 0.1-Da fragment mass tolerance, and a maximum of three dynamic modifications per peptide. False discovery rates (FDRs) for peptide spectral matches (PSMs) were calculated and filtered using the Percolator node in the Proteome Discoverer. The Protein FDR Validator node was used to calculate q values for proteins based on the false discovery rates calculated by searching against the target-decoy database. Proteins with q values of < 0.01 were categorized as high-confidence and those with q-values between 0.01 – 0.05 were categorized as medium-confidence. We combined all search results into a multiconsensus report and proteins were filtered to retain only those with an overall FDR of 5% or less. Normalized spectral abundance factors (NSAFs) were calculated for each bacterial and animal species (OrgNSAF) and multiplied by 100 to represent relative protein abundance as a percentage.

1. Jensen M, Wippler J, Kleiner M. Evaluation of RNAlater as a field-compatible preservation method for metaproteomic analyses of bacterium-animal symbioses. *Microbiol Spectr* 2021;9:e0142921. https://doi.org/10.1128/Spectrum.01429-21

Supplementary Tables :

Table S1: MAG coverage statistics calculated using bbmap.sh

Table S2: 426 publicly available genomes that passed the dereplicate filter and checkM prior to alignment and construction of the phylogenomic tree in Figure 1

Table S3: Metadata input for pangenome in Figure 2 including sample collection information when available and GTDB repository identification for free-living outgroup

Table S4: Anvio functional enrichment performed analysis of Kegg metabolic modules performed on the pangenome analysis represented in Figure 2

Table S5: Anvio functional enrichment performed analysis of COG20 annotated functions performed on the pangenome analysis represented in Figure 2

Table S6: MEBS tool output for presence/absence clustering filtered based on presence in 100% of symbiont genomes and absence in 100% of genomes in the comparison cluster column

Table S7: MEBS output marker gene set completeness values for all genomes included in MEBS analysis

Table S8: MEBS analysis performed for figure 2 concatenated with the metadata table from Langwig et al. to show corresponding clusters and their degree of alignment. *Desulfoconcordia* genomes and free-living relatives in cluster7 are included at the end of the table as they were not included in Langwig et al.

Table S9: Total protein identifications per taxa in bulk metaproteome dataset - proxy for biomass contributions

Table S10: Organism normalized Desulfoconcordia (Delta4) data extracted from bulk metaproteomes

Table S11: Matching of symbiont-enriched COG20_Function annotation from Anvi'o to metaproteome abundance data for symbiont enrichment samples (filtered for those COG20 Functions present in at least 80% of symbiont genomes and absent in all of the GTDB relatives)

Table S12: Desulfoconcordia proteomes extracted from single worm metaproteomes - reanalyzed data downloaded from PRIDE; accession: PXD014591

Supplementary Figures :

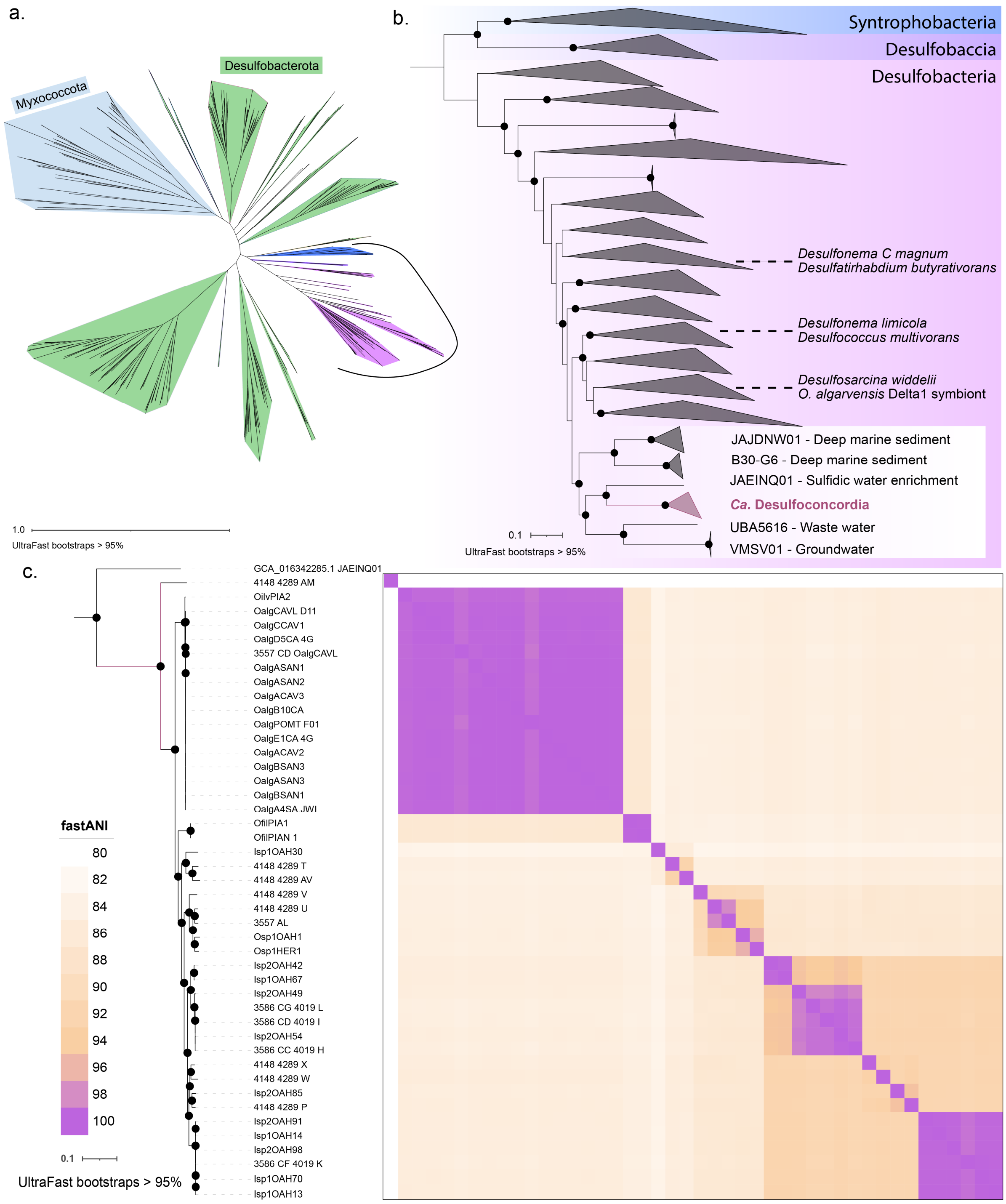

Figure S1: Phylogenomic placement of *Desulfoconcordia* symbionts. The *Desulfoconcordia* symbiont MAGs are displayed in their phylogenomic context according to the GToTree workflow for GTDB reference genomes. a) Phylogenomic tree generated with

426 publicly available genomes and 14 *Desulfoconcordia* MAGs places these symbionts in the Desulfobacteria class. b) For a more detailed view, the classes Syntrophobacteria and Desulfobaccia were used as outgroups for the class Desulfobacteria. Selected representative type strain members of Desulfobacteria are highlighted in their respective clades. Close representatives to the monophyletic *Desulfoconcordia* symbiont clade are labeled with their GTDB taxonomic genus-level identifiers. Black dots represent UltraFast bootstrap scores greater than 95%. c) Average nucleotide identity scores calculated using fastANI with a color scale delineation representing the 95% cutoff for species distinction.

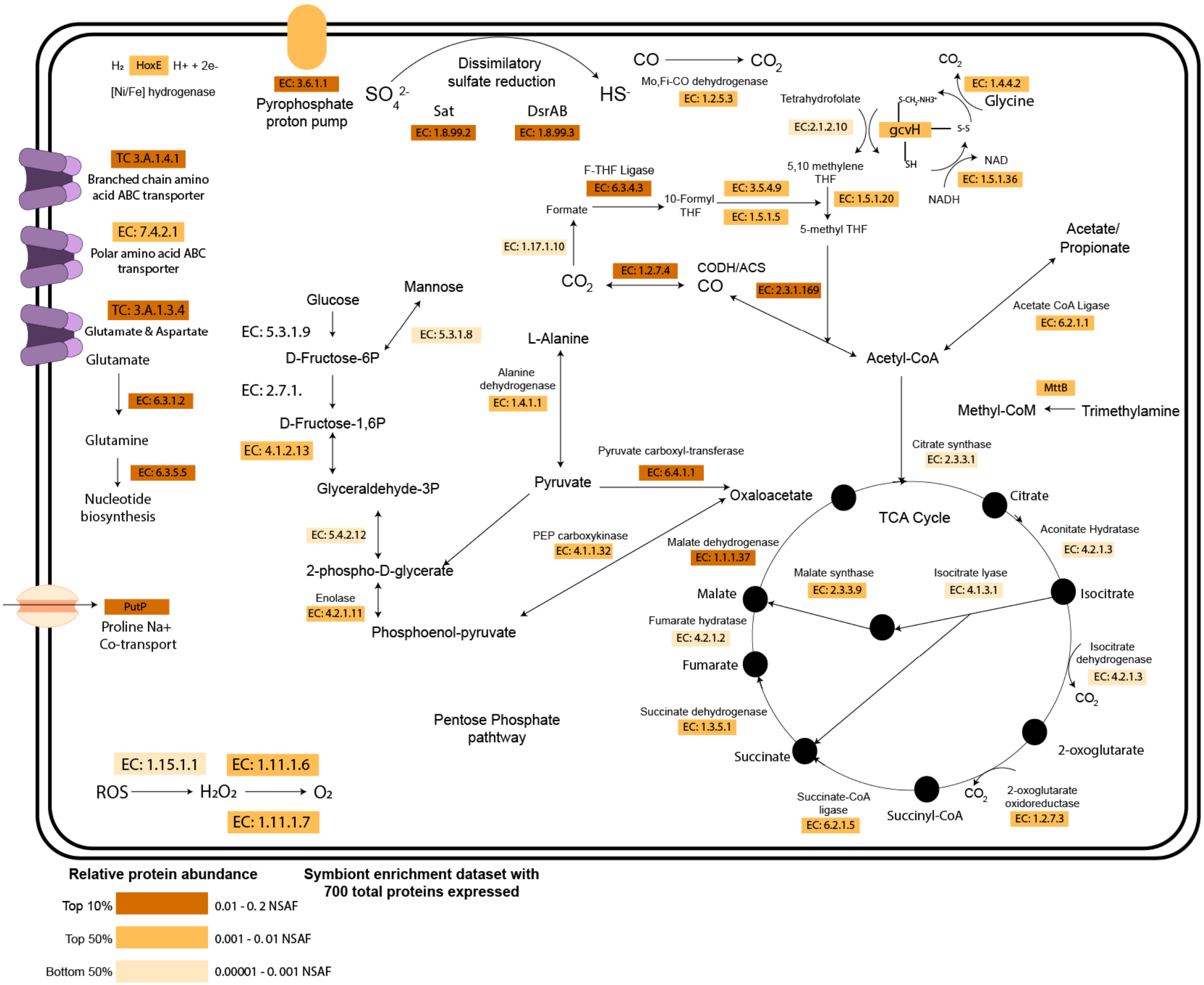

Figure S2: Detailed metabolic overview of *Desulfoconcordia*. A metabolic reconstruction of the pathways from the simplified metabolic overview based on enrichment samples (n=50 worms homogenized). EC numbers or gene names of key enzymes in each pathway are highlighted in different shades of orange to represent their respective relative abundance within the metaproteome.

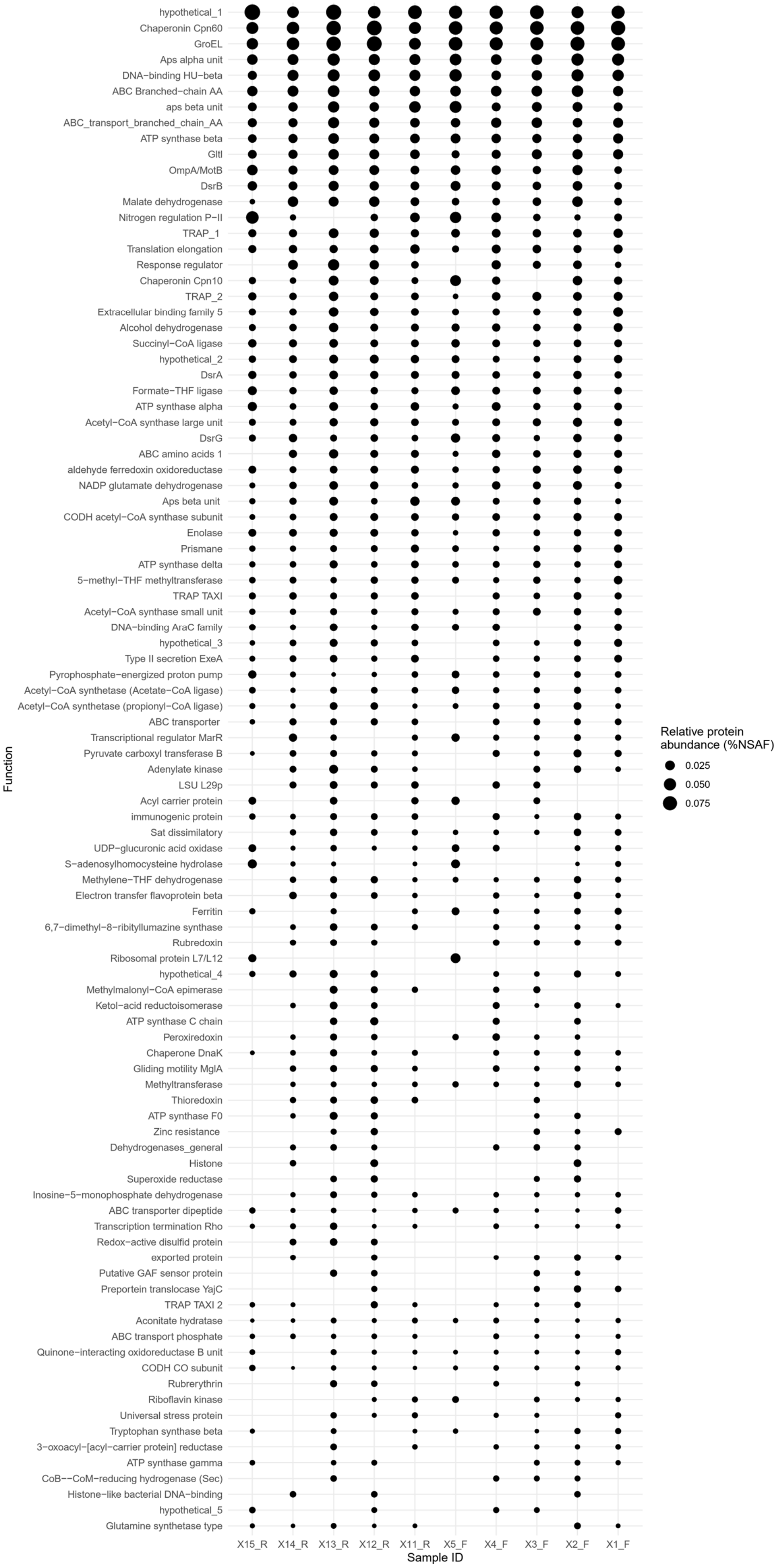
hypothetical_1
Chaperonin Cpn60
GroEL
Aps alpha unit
DNA−binding HU−beta
ABC Branched−chain AA
aps beta unit
ABC_transport_branched_chain_AA
ATP synthase beta
Gltl
OmpA/MotB
DsrB
Malate dehydrogenase
Nitrogen regulation P−II
TRAP_1
Translation elongation
Response regulator
Chaperonin Cpn10
TRAP_2
Extracellular binding family 5
Alcohol dehydrogenase
Succinyl−CoA ligase
hypothetical_2
DsrA
Formate−THF ligase
ATP synthase alpha
Acetyl−CoA synthase large unit
DsrG
ABC amino acids 1
aldehyde ferredoxin oxidoreductase
NADP glutamate dehydrogenase
Aps beta unit
CODH acetyl−CoA synthase subunit
Enolase
Prismane
ATP synthase delta
5−methyl−THF methyltransferase
TRAP TAXI
Acetyl−CoA synthase small unit
DNA−binding AraC family
hypothetical_3
Type II secretion ExeA
Pyrophosphate−energized proton pump
Acetyl−CoA synthetase (Acetate−CoA ligase)
Acetyl−CoA synthetase (propionyl−CoA ligase)
ABC transporter
Transcriptional regulator MarR
Pyruvate carboxyl transferase B
Adenylate kinase
LSU L29p
Acyl carrier protein
immunogenic protein
Sat dissimilatory
UDP−glucuronic acid oxidase
S−adenosylhomocysteine hydrolase
Methylene−THF dehydrogenase
Electron transfer flavoprotein beta
Ferritin
6,7−dimethyl−8−ribityllumazine synthase
Rubredoxin
Ribosomal protein L7/L12
hypothetical_4
Methylmalonyl−CoA epimerase
Ketol−acid reductoisomerase
ATP synthase C chain
Peroxiredoxin
Chaperone DnaK
Gliding motility MglA
Methyltransferase
Thioredoxin
ATP synthase F0
Zinc resistance
Dehydrogenases_general
Histone
Superoxide reductase
Inosine−5−monophosphate dehydrogenase
ABC transporter dipeptide
Transcription termination Rho
Redox−active disulfid protein
exported protein
Putative GAF sensor protein
Preportein translocase YajC
TRAP TAXI 2
Aconitate hydratase
ABC transport phosphate
Quinone−interacting oxidoreductase B unit
CODH CO subunit
Rubrerythrin
Riboflavin kinase
Universal stress protein
Tryptophan synthase beta
3−oxoacyl−[acyl−carrier protein] reductase
ATP synthase gamma
CoB−−CoM−reducing hydrogenase (Sec)
Histone−like bacterial DNA−binding
hypothetical_5
Glutamine synthetase type
Function
X15_R
X14_R
X13_R
X12_R
X11_R
X5_F
X4_F
X3_F
X2_F
X1_F
Sample ID
Relative protein abundance (%NSAF)
0.025
0.050
0.075

Figure S3: Top 100 expressed *Desulfoconcordia* genes in single *O. algarvensis* individuals. A reanalysis of published metaproteomes (Methods) showing the top 100 abundant proteins across 10 host individuals (**Table S12**).
